# Rethinking How to Promote Maternity Care-Seeking: Factors Associated With Institutional Delivery in Guinea

**DOI:** 10.1080/07399332.2014.916293

**Published:** 2014-07-01

**Authors:** Ellen Brazier, Renée Fiorentino, Saidou Barry, Yaya Kasse, Sita Millimono

**Affiliations:** ^a^ EngenderHealth, New York, New York, USA; ^b^ EngenderHealth, Conakry, Guinea

## Abstract

This article presents findings from a study on women's delivery care-seeking in two regions of Guinea. We explored exposure to interventions promoting birth preparedness and complication readiness among women with recent live births and stillbirths. Using multivariate regression models, we identified factors associated with women's knowledge and practices related to birth preparedness, as well as their use of health facilities during childbirth. We found that women's knowledge about preparations for any birth (normal or complicated) was positively associated with increased preparation for birth, which itself was associated with institutional delivery. Knowledge about complication readiness, obstetric risks, and danger signs was not associated with birth preparation or with institutional delivery. The study findings highlight the importance of focusing on preparation for all births—and not simply obstetric emergencies—in interventions aimed at increasing women's use of skilled maternity care.

While there is consensus on skilled attendance during childbirth as an important intervention for improving maternal survival, rates of skilled attendance remain low, particularly in sub-Saharan Africa, and evidence is lacking on effective interventions for increasing women's access to and use of these services. Birth preparedness/complications (BP/CR) interventions offer an intuitively appealing strategy for increasing women's use of institutional delivery care. Evidence on the effectiveness of these interventions, however, is mixed. In this article, we explore women's knowledge related to both birth preparedness and complications readiness, as well as the association of each with care-seeking preparations and behaviors. We will suggest that increased attention should be given during antenatal consultations and through community interventions to promoting and supporting preparation for birth—not simply raising awareness about danger signs and complication readiness.

While global and regional estimates of maternal mortality have been the focus of considerable debate in recent years (Byass & Graham, 2011; Hogan et al., 2011; Lozano et al., 2011; World Health Organization [WHO], 2010), there is agreement on the essential strategies for improving maternal health outcomes. Increasing access to skilled care during childbirth and during obstetric emergencies—along with access to family planning and safe abortion care—were recently reaffirmed as critical for sustaining progress made since the launch of the Millennium Development Goals (MDGs) in 2000 and for addressing the “unfinished business” of the fifth MDG to improve maternal health (Langer, Horton, & Chalamilla, 2013).

Despite consensus about priority maternal health interventions, evidence is lacking on how best to increase use of skilled maternity care for the approximately 144 million women who give birth each year. In sub-Saharan Africa, rates of skilled attendance have changed little since the launch of the MDGs in 2000 (United Nations [UN], 2012). Researchers analyzing data from 54 Countdown to 2015 countries have shown that wealth inequalities remain far greater for skilled attendance at birth than any of the other proven interventions for maternal, newborn, and child health for which national data are available (Barros et al., 2012).

Initiatives promoting birth preparedness and complication readiness (BP/CR) have been described as “one of the conceptually compelling and logical means” of ensuring timely receipt of skilled and emergency obstetric care (Stanton, 2004). In the extant literature, however, operational definitions of BP/CR have varied widely, contributing to a diversity of approaches and little consistent evidence of intervention effectiveness or on essential elements of these interventions; some researchers have focused primarily on preparations for obstetric emergencies (e.g., heightening awareness of danger signs, identifying a facility where emergency obstetric care is available, setting aside money for an emergency, identifying a potential blood donor, and arranging for emergency transport; McPherson, Khadka, Moore, & Sharma, 2006; Moran et al., 2006; Mutiso, Qureshi, & Kinuthia, 2008). Other researchers have explored both complication readiness and planning for normal delivery without making a clear distinction between the two (Agarwal, Sethi, Srivastava, Jha, & Baqui, 2010; Ekabua et al., 2011; Hailu, Gebremariam, Alemseged, & Deribe, 2011; Kabakyenga, Ostergren, Turyakira, & Pettersson, 2012; Kakaire, Kaye, & Osinde, 2011; Magoma et al., 2013; Mullany, Becker, & Hindin, 2007; Turan, Tesfagiorghis, & Polan, 2011), and several have included women's use of antenatal care (ANC), the content of those consultations, or both, among measures of birth preparedness (Kakaire et al., 2011; Karkee, Lee, & Binns, 2013; McPherson et al., 2006).

In a recent cluster randomized trial in rural Tanzania, use of institutional delivery care was found to be higher in intervention clusters where ANC providers offered individualized support to pregnant women in developing a birth plan to prepare for delivery and possible complications (Magoma et al., 2013). Similarly, in a prospective cohort study in one district of Nepal, Karkee and colleagues (2013) found that the number of birth preparations made during pregnancy was positively associated with use of institutional delivery care. Evidence on the association between BP/CR and women's care-seeking during childbirth from other research, however, is inconclusive. The results of other intervention studies, which used measures of BP/CR such as women's knowledge about danger signs/risks, their use of antenatal care during pregnancy, and their preparation for newborn care and items for the newborn, showed that there was weak or no association between BP/CR interventions and women's use of skilled maternity care (Kumar et al., 2008; McPherson et al., 2006; Mullany et al., 2007).

In the face of existing evidence, it remains unclear which elements of birth preparedness and complications readiness interventions are important in supporting and motivating women to seek institutional delivery care during birth. In this article, we investigate this question further by differentiating between women's knowledge about birth preparations and their knowledge about complications readiness and obstetric risks, while exploring factors associated with their levels of preparation for a recent birth and their use of institutional delivery care. Using a subset of data from an evaluation study conducted in Guinea, we explore the influence of exposure to BP/CR messages from two sources (antenatal care consultations and community-level sources) on women's knowledge related to birth preparedness and complications readiness, their levels of preparation for childbirth, and, ultimately, their care-seeking during delivery (see Figure 1).

**FIGURE 1:**
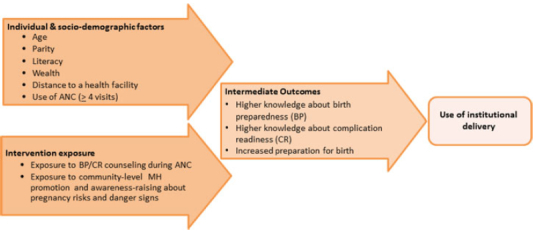
Conceptual model of causal pathway between intervention exposure, intermediate outcomes, and behavioral outcomes.

## METHODS

### Study Area

Located in West Africa, the Republic of Guinea is one of the world's poorest countries, ranked 178th out of 187 countries on the 2011 Human Development Index (United Nations Development Program [UNDP], 2011). Estimates of maternal mortality are high, ranging from 610 to 860 maternal deaths per 100,000 live births (Hogan et al., 2011; Lozano et al., 2011; WHO, 2010). Women's use of institutional delivery is low (46% nationally).

The study was conducted mid-2011 in two prefectures of Guinea: Kissidougou prefecture in Faranah Region in southeastern Guinea, and Labé prefecture in Labé region in the north of the country. Both regions of the country have poor maternal health indicators, with only 29.0% and 26.9% of births, respectively, taking place in health facilities (Institute National de la Statistique [INS], 2012).

Selected villages in Kissidougou and Labé prefectures had been the focus of a 5-year project to prevent and address obstetric fistula. Funded by USAID and led by EngenderHealth, the Fistula Care project focused on increasing local surgical capacity to repair fistula and on the prevention of obstetric fistula through improved labor monitoring at selected health facilities and through supporting village safe motherhood committees (VSMCs) to promote the use of skilled care throughout pregnancy and childbirth. Established in selected periurban communities in each prefecture, the VSMCs were trained to raise awareness about obstetric risks, danger signs, and causes of obstetric fistula and to promote the use of ANC during pregnancy and institutional delivery care during childbirth. The volunteers serving on the VSMCs received training on these topics, as well as a flipchart for use in leading health education sessions at the community level and in conducting household-level pregnancy monitoring visits. The community-level intervention was launched in Kissidougou in late 2007 and expanded to Labé in 2009. After the initial establishment and training of the VSMCs, quarterly review meetings were conducted for all members of the VSMCs in each region to reinforce volunteers’ skills and knowledge about maternal health and to provide a forum for each committee to report on their awareness-raising and pregnancy-monitoring activities.

### Study Design and Sample

Data were collected through a population-based household survey of women of reproductive age in 30 villages across the two regions as part of a retrospective evaluation of the community-level intervention. In each village, households were randomly selected for interview using household lists developed by the National Institute of Statistics for the 2011 national census. In each household, an interview was conducted with the household head to gather information on all household residents and household assets. In addition, up to two women of reproductive age (15–49 years) were interviewed.

Interviews with women focused on their knowledge about maternal health and BP/CR and their perceptions of community norms related to these topics. Basic demographic information on respondents’ schooling, literacy, employment, ethnicity, and religion were also collected. Among women who had had a live birth or stillbirth within the 5 years prior to the study, questions explored their exposure to community- and facility-level interventions promoting maternal health and BP/CR, as well as their birth preparedness practices and their care-seeking during pregnancy, delivery, and the postpartum period.

### Ethical Considerations

The Comité National d’Ethique pour la Recherche en Santé of Guinea (National Ethics Committee for Health Research) reviewed the study protocol and survey tools and provided ethical clearance for the study. Informed consent was obtained from all survey respondents.

### Data Collection and Management

Data collection and entry were conducted by a consultant research firm, StatView International, based in Conakry. Data collection took place over a 6-week period between July and August 2011. At least two attempts were made to interview selected households, and a total of 1,846 households were successfully interviewed. A total of 2,335 women of reproductive age were interviewed, of whom 1,333 had given birth within the previous 5 years, and 763 had given birth within the past 24 months.

In view of the potential for recall errors regarding preparation for births to increase with time (Solnes Miltenburg, et al., 2013; Stanton, 2004), our analysis of factors associated with birth preparedness and use of maternal health services focused on women who had given birth with the past 24 months.

### Variables in the Study

Given that women attending health facilities do not have the ability to influence the type or qualification of the service provider on duty, we defined institutional delivery (as opposed to skilled attendant) as the key outcome variable for measuring maternal health care-seeking behaviors. Institutional delivery was defined as giving birth in a hospital, health center, or health post (public or private/mission).

Other intermediate outcome variables were created to measure women's knowledge related to maternal health and their level of preparation for their most recent birth. We created two knowledge indexes to measure women's knowledge related to birth preparedness and complication readiness. The Birth Preparedness (BP) Knowledge Index measured women's knowledge about important birth preparations for any delivery (as opposed to preparations specifically for an emergency). The BP Knowledge Index was based on whether respondents cited the following as important preparations for birth: deciding on the place of delivery; discussing delivery plans with their husband/family members; saving money for delivery; making arrangements for transport to the place of delivery; and obtaining “approval” for delivery plans from household decisionmakers. Women received a score of 1 for each birth preparation that they were able to mention spontaneously. A cut-off value of 2 (i.e., ≥ 2) was used to create a dichotomous variable for “high” vs. “low” individual knowledge about birth preparedness.

The Complications Readiness (CR) Knowledge Index measured women's knowledge about risks and danger signs related to pregnancy and childbirth, and their knowledge about preparedness for obstetric emergencies. It was based on whether respondents agreed that every pregnancy was risky; knew at least three danger signs during pregnancy and childbirth; knew at least three danger signs during the postpartum period; and mentioned identifying a possible blood donor among important preparations for birth. A score between 0 and 4 was assigned to each woman, and a cut-off value of ≥ 3 was used to create a dichotomous variable for “high” vs. “low” individual knowledge related to CR.

The Birth Preparedness Behavioral Index measured the number of preparations that women reported (unprompted) that they had made for their most recent birth, including whether they had discussed institutional delivery with their partner; discussed transport; discussed how to pay for the delivery; identified a possible blood donor; or set aside money for the delivery. A score between 0 and 4 was assigned to each woman, based on her responses, and a cut-off value of ≥ 3 was used to create a dichotomous variable for “high” vs. “low” BP.

Two composite variables were created to allow for exploration of the association between intervention factors and the dependent variables described above. Because the clinical and counseling content of ANC has been shown to influence care-seeking during childbirth (Barber 2006; Bloom, Lippeveld, & Wypij, 1999; Mpembeni et al., 2007), a composite variable was developed to summarize the counseling provided to women during their ANC visits. The BP/CR Counseling Index was based on whether women reported that, during any of their ANC visits, they were advised to deliver at a health facility; advised about danger signs during pregnancy and delivery; and advised on any of the following delivery preparations: saving money, arranging for transport, discussing delivery plans with family members, or identifying a blood donor. Based on their responses to these questions, scores between 0 and 3 were assigned to each woman, and a cut-off value of ≥ 2 was used to create a dichotomous variable for “high” vs. “low” exposure to counseling on birth preparedness.

A second intervention exposure index was the Community Exposure Index, which measured each woman's exposure to community-level maternal health promotion activities carried out by the VSMCs, local health and hygiene committees, or other community-level health agents. The Community Exposure Index was derived based on whether a woman agreed that there was a local committee involved in promoting maternal health through community discussions and pregnancy monitoring visits; mentioned (unprompted) a community health committee or agent as a main source of maternal health information and help; and had personally attended a community discussion on maternal health during the past year. Based on their responses to these questions, scores between 0 and 3 were assigned to each woman, and a cut-off value of ≥ 2 was used to create a dichotomous variable for “high” vs. “low” exposure to community-level maternal health promotion activities.

Principal components analysis (Filmer & Pritchett, 2001) was used to compute wealth quintiles based on data related to household assets, which included consumer items (e.g., radio, television, bicycle, etc.) and dwelling characteristics (flooring materials, type of drinking water source, toilet facilities, etc.). Each household was assigned a standardized score for each asset, with the score determined by household ownership of that asset. Scores were summed for each household, and individuals were ranked according to the total score of the household in which they resided. The full sample (2,335 women) was divided into population quintiles, which represent the poorest 20% of the population, second poorest 20%, middle 20%, fourth poorest 20%, and least poor 20% of the population, respectively.

### Statistical Analysis

Data were analyzed in SPSS (version 20.0). Descriptive analyses and frequencies were run for all variables of interest. Bivariate analyses were performed to explore the association of intervention exposure and sociodemographic factors with (a) intermediate knowledge outcomes related to birth preparedness and obstetric risks and (b) behavioral outcomes related to birth preparedness and delivery in a health facility.

Multivariate logistic regression models were used to explore the association between intervention exposure and intermediate knowledge outcomes on behavioral outcomes of interest while controlling for known predictors (wealth, literacy, distance to a health facility, age, and parity). A cut-off value of *p* < 0.1 was used as the criterion for including predictor variables in the multivariate regression models.

## RESULTS

Characteristics of the women in our sample are shown in Table 1. Just over half of study participants (52%) were residents of Kissidougou region, while 47% were from Labé. The mean age of women in the sample was 28 years (+/−7 years), and mean parity was 3.9. Almost all study participants were married, and the majority had never attended school. Literacy was low, with 79% reporting that they could not read at all; only 14% reported that they could read easily. Distances to health facilities that provided delivery care were relatively small, with almost half of the women reporting that they lived within 2 kilometers of such a facility.

**TABLE 1:** Participant Characteristics (*N* = 763)

Characteristic	Mean	Standard deviation
Age (in years)	28.2	7.4
Age at time of most recent birth (in years)	27.3	7.4
Wealth quintile	3.0	1.4
Parity	3.9	2.3
Characteristic	Number	Percent (%)
Region		
Kissidougou	401	52.6
Labé	362	47.4
Religion		
Muslim	620	81.3
Christian	143	18.7
Any education	243	31.8
Literacy (can read with difficulty or easily)	157	20.6
Currently married	719	94.2
Wealth quintile		
Poorest	161	21.1
Second poorest	153	20.1
Middle	147	19.3
Fourth poorest	161	21.1
Least poor	139	18.2
Employed in remunerated activity	468	61.4
Member of community group	418	54.8
Ever travelled outside village	187	24.5
Live within 2 km of maternity care facility	366	48.0
Live more than 5 km of maternity care facility	169	22.1
Live within 30 minutes of maternity care facility	315	41.3
BP/CR Counseling Index (High)	309	44.8
Community Exposure Index (High)	304	39.8
Complication Readiness (CR) Knowledge Index (High)	108	14.2
Birth Preparedness (BP) Knowledge Index (High)	237	31.1
Birth Preparedness Behavioral Index (High)	262	34.3
At least one ANC visit	616	80.7
At least four ANC visits	434	57.0
Delivered in a health facility	396	51.9

Almost half (45%) of the women in the sample had a high score on the BP/CR Counseling Index, meaning that, during their most recent pregnancy, they recalled receiving more comprehensive counseling on place of delivery, important birth preparations, and obstetric risks and danger signs. Similarly, 40% had a high score on the Community Exposure Index, indicating that they had personally been exposed to community-level awareness-raising sessions about maternal health risks, pregnancy monitoring visits, or both conducted by community health cadres or volunteers.

Only 31% of respondents had a high score on the BP Knowledge Index, and only 14% had a high score on the CR Knowledge Index. Thirty-four percent of women in our sample had made three or more preparations for their most recent birth, and had a high score on the BP Behavioral Index. The majority of respondents (81%) had attended ANC at least once during their most recent pregnancy, and 57% had attended at least four ANC visits. Just over half (52%) had delivered in a health facility.

Results of bivariate analyses exploring the association of selected sociodemographic and intervention factors with high scores on the BP and CR Knowledge Indexes are shown in Table 2. Both intervention exposure indexes (i.e., the BP/CR Counseling Index and the Community Exposure Index) were positively and significantly associated with high scores on the two knowledge indexes. Few of the sociodemographic variables were associated with high scores on either knowledge index, with the exception of wealth, which was inversely related to knowledge. Literacy was significantly associated with knowledge about birth preparedness, but not associated with CR knowledge. Both intervention exposure indexes were positively associated with high scores on the BP Behavioral Index (see Table 3). The BP/CR Counseling Index was positively associated with institutional delivery, whereas the association between exposure to community-level maternal health promotion activities and institutional delivery was borderline in significance (*p* = 0.07). A high score on the BP Knowledge Index was significantly associated with institutional delivery; however, no such association was observed for the CR Knowledge Index.

**TABLE 2:** Bivariate Associations of Sociodemographic, Individual, and Intervention Factors With BP and CR Knowledge

	BP knowledge (high) *N* = 763		CR knowledge (high) *N* = 763	
Characteristic	*N* (%)	OR (95% CI)	*N* (%)	OR (95% CI)
Age (≥ 25 years)	160 (0.9)	1. 1 (0.8, 1.5)	76 (10.0)	1.3 (0.8, 1.9)
Literacy	45 (5.9)	1.7 (1.1, 2.5)*	14 (1.8)	0.9 (0.5, 1.6)
Parity (≥ 2)	207 (27.1)	1.4 (0.9, 2.3)	91(11.9)	1.0 (0.6, 1.8)
Wealth (wealthiest two quintiles)	77 (10.1)	0.7 (0.5, 0.9)**	27 (3.5)	0.5 (0.3, 0.7)**
Distance to health facility ≤ 2 km	115 (15.1)	1.0 (0.7, 1.3)	47 (6.2)	0.7 (0.5, 1.1)
At least 1 ANC visit	186 (24.4)	1.0 (1.0, 1.0)	95 (12.4)	0.4 (0.0, 4.1)
At least 4 ANC visits	145 (19.0)	1.3 (1.0, 1.8) *p* =.09	62 (8.1)	1.0 (0.7, 1.5)
BP/CR Counseling Index (High)	120 (15.7)	2.0 (1.4, 2.7)***	62 (8.1)	2.4 (1.5, 3.7)***
Community Exposure Index (High)	130 (17.0)	2.5 (1.8, 3.4)***	56 (7.3)	1.8 (1.2, 2.7)**

*p ≤.05; **p ≤.01; ***p ≤.001.

**TABLE 3:** Bivariate Associations of Sociodemographic, Individual, Intervention, and Intermediate Outcome Variables With Birth Preparedness and Institutional Delivery

	Birth preparedness (high) *N* = 763		Institutional delivery *N* = 763	
Characteristic	*N* (%)	OR (95% CI)	*N* (%)	OR (95% CI)
Age (≥ 25 years)	176 (23.1)	1.1 (0.8, 1.5)	257 (33.7)	0.9 (0.7, 1.2)
Literacy	44 (5.7)	1.4 (0.9, 2.2)	111 (14.5)	2.7 (1.9, 4.0)***
Parity (≥ 2)	223 (29.2)	1.1 (0.7, 1.7)	326 (42.7)	0.8 (0.5, 1.1)
Wealth (wealthiest two quintiles)	107 (14.0)	1.1 (0.5, 1.6)	185 (24.2)	1.9 (1.4, 2.6)***
Distance to health facility ≤ 2 km	131 (17.2)	1.1 (0.8, 1.6)	234 (30.7)	2.8 (2.1, 3.8)***
At least 1 ANC visit	211 (27.7)	1.0 (0.9, 1.0)	309 (40.4)	1.0 (0.9, 1.0)
At least 4 ANC visits	166 (21.8)	1.7 (1.2, 2.3)**	243 (31.8)	1.5 (1.1, 1.9) **
BP/CR Counseling Index (High)	159 (20.8)	4.7 (3.3, 6.6)***	196 (25.7)	2.4 (1.7, 3.2)***
Community Exposure Index (High)	134 (17.6)	2.0 (1.5, 2.8)***	170 (22.3)	1.3 (1.0, 1.8) *p* =.07
CR Knowledge Index (High)	45 (5.9)	1.4 (0.9, 2.1)	61 (8.0)	1.2 (0.8, 1.9)
BP Knowledge Index (High)	126 (16.5)	3.0 (2.2, 4.2)***	145 (19.0)	1.7 (1.3, 2.4)**
BP Behavioral Index (High)	—	—	184 (24.1)	3.6 (2.6, 5.0)***

*p ≤.05; **p ≤.01; ***p ≤.001.

We used multivariate logistic regression to assess the association between intervention exposure and intermediate knowledge outcomes on (a) birth preparedness and (b) institutional delivery, while controlling for individual and sociodemographic factors (e.g., literacy, wealth, and distance to a health facility) that were significantly associated with the respective outcomes of interest in our bivariate analyses. We controlled for high ANC attendance (≥ 4 visits) in view of the fact that women who attend ANC more frequently may be more inclined to seek professional care during delivery.

We present multivariate regression results for birth preparedness in Table 4. Because none of the sociodemographic or individual variables (e.g., wealth, literacy, parity, or age) met specified criteria (*p* < 0.1) for inclusion in the model, they were excluded, along with the CR Knowledge Index. In the first model, in which we controlled for increased use of ANC (≥ 4 visits), receipt of counseling on BP and exposure to community-level maternal health promotion activities were both significantly associated with higher preparation for childbirth. In a second model, the BP Knowledge Index was added. The effect of the Community Exposure Index was attenuated in the expanded model; however, the BP/CR Counseling Index remained positively and significantly associated with increased preparation for childbirth, as did a high score on the BP Knowledge Index and receipt of at least four ANC visits (see Table 4).

**TABLE 4:** Multivariate Regression Results—Factors Associated With Increased Birth Preparedness

Variables (reference category)	Model 1 adjusted OR (95% CI)	Model 2 adjusted OR (95% CI)
Intervention exposure variables		
BP/CR Counseling Index (high vs. low score)	4.3 (3.0, 6.1)***	4.0 (2.8, 5.8)***
Community Exposure Index (high vs. low score)	1.6 (1.1, 2.3)*	1.4 (1.0, 2.0)
ANC use (≥ 4 ANC visits during pregnancy vs. < 4 ANC visits)	1.6 (1.1, 2.3)*	1.6 (1.1, 2.3)*
Intermediate outcome variables		
BP Knowledge Index (high vs. low score)	—	2.2 (1.5, 3.2)***

*p ≤.05; **p ≤.01; ***p ≤.001.

Similar analyses were performed using institutional delivery as the outcome variable (Table 5). In the first model, the two intervention exposure variables were included, along with sociodemographic and individual variables that met criteria for inclusion. The BP Knowledge Index and the BP Behavioral Index were included in an expanded model to explore their association with institutional delivery (Model 2). In this expanded model, exposure to BP/CR Counseling during ANC remained significantly associated with institutional delivery, along with wealth, literacy, and distance to a health facility. Women's level of birth preparation was also significantly associated with institutional delivery in the full model; women making at least three preparations for childbirth were twice as likely to deliver at a facility as those who made fewer birth preparations. The effect of women's knowledge about birth preparedness was attenuated and not significant, however, a finding that is to be expected given the strong positive association between birth preparedness knowledge and levels of birth preparedness (see Table 4).

**TABLE 5:** Multivariate Regression Results: Factors Associated With Institutional Delivery

Variables (reference category)	Model 1 adjusted OR (95% CI)	Model 2 adjusted OR (95% CI)
Intervention exposure variables		
BP Counseling Index (high vs. low score)	2.3 (1.6, 3.2)***	1.8 (1.2, 2.6)**
Community Exposure Index (high vs. low score)	1.3 (0.9, 1.9)	1.1 (0.8, 1.7)
Sociodemographic & individual variables		
Wealth (two wealthiest quintiles vs. three poorest quintiles)	1.9 (1.3, 2.7)**	2.0 (1.4, 3.0)**
Literacy (able to read easily or with difficulty vs. cannot read at all)	2.4 (1.5, 4.1)**	2.4 (1.4, 4.3)**
Distance (≤ 2 km of maternity care vs. > 2 km)	2.6 (1.8, 3.6)***	2.7 (1.9, 3.9)***
ANC use (≥ 4 ANC visits during pregnancy vs. < 4 ANC visits)	1.4 (1.0, 1.9)	1.3 (0.9, 1.8)
Intermediate outcome variables		
BP Knowledge Index (high vs. low score)	—	1.3 (0.9, 2.0)
BP Behavioral Index (high vs. low score)	—	2.5 (1.7, 3.8)***

*p ≤.05; **p ≤.01; ***p ≤.001.

### Study Limitations

This study has several limitations. It has been noted by Stanton (2004) that women's retrospective self-reported preparations for childbirth may be influenced by the care they receive during childbirth, and that such self-reports may be subject to increased recall errors as time elapses. We had no means of independently verifying that reported preparations had been made for the births under question. In addition, it is important to acknowledge that women's knowledge related to birth preparedness and obstetric complications may be informed by experiences during their most recent pregnancy or birth. Because this study used a retrospective design, we cannot be sure that respondents’ BP/CR knowledge at the time of the survey was consistent with their knowledge at the time of the birth in question.

Third, the study did not include assessments of the capacity of local health facilities and maternity staff to provide care for normal and complicated deliveries. Clients’ perceptions of service quality are known to influence their care-seeking behaviors; however, we were not able to control for service quality across the study areas. Finally, as noted earlier, the study was conducted in two periurban areas of Guinea, and the sample is not nationally representative.

## DISCUSSION

Interventions promoting BP/CR are intuitively appealing; however, the influence of such interventions on women's preparation for birth and their use of institutional delivery care is unclear. To date, in program design and intervention research, operational definitions of birth preparedness have varied widely, with some such interventions focused primarily on preparations for any delivery (normal or complicated) and other interventions focusing primarily on readiness for both maternal and newborn complications. Research and evaluations related to BP/CR interventions have yielded mixed evidence on the efficacy of such interventions; while some studies have indicated that BP/CR interventions are effective in increasing use of skilled delivery care (Magoma et al., 2013), others—and particularly studies of interventions that focused primarily on complications readiness—have not shown any positive association with institutional delivery (Kumar et al., 2008; McPherson et al., 2006). Nonetheless, many researchers call for increased attention to educating pregnant women about obstetric risks and danger signs in order to increase their use of skilled maternity care and to prevent delays in care-seeking for complications (Ekabua et al., 2011, Hailu et al., 2011, Kabakyenga et al., 2012; Kakaire et al., 2011; Mutiso et al., 2008).

The findings of this study help to further elucidate the pathway between BP/CR interventions, household preparation for birth, and women's use of institutional delivery care. We found that facility- and community-level interventions promoting BP/CR were both positively associated with increased knowledge about both birth preparedness and complication readiness. Complication readiness knowledge (i.e., knowledge about obstetric risks and danger signs), however, was not associated with increased preparation for childbirth or with women's use of health facilities for delivery.

In contrast, women's knowledge about routine birth preparations (e.g., deciding place of delivery, saving money for delivery, making arrangements for transport, etc.) was positively associated with their practice of birth preparedness, which was itself strongly associated with institutional delivery. Importantly, neither literacy nor wealth status were significantly associated with higher preparation for birth. In view of the strong association between birth preparation and use of institutional delivery, these findings are noteworthy as they suggest that promoting birth preparedness among poor and low-literate women can be an important strategy for addressing wealth disparities in use of professional maternity care.

Other researchers (Barber, 2006; Bloom, et al., 1999; Kabakyenga et al., 2012; Mpembeni et al., 2007) have highlighted the positive relationship between the quantity of ANC (measured in terms of number of visits) and women's use of institutional delivery care. Our findings underscore the importance of the counseling that women receive during these visits. In our multivariate models, receiving BP/CR counseling had a stronger association with women's preparation for birth and their use of institutional delivery than did receipt of four or more ANC visits.

The above findings have important implications for the design of both community- and facility-level interventions to improve maternal survival. During the past two decades, considerable efforts have been made in community-level interventions to raise awareness of pregnancy-related risks and danger signs in order to address the “three delays” (Thaddeus & Maine, 1994) that contribute to maternal mortality and morbidity. A key assumption behind such interventions is that knowledge about obstetric risks and danger signs will motivate the use of professional maternity care and will reduce delays in recognizing life-threatening obstetric complications when they arise and in reaching and receiving appropriate care. In our study, exposure to community-level maternal health promotion efforts was associated with increased knowledge about obstetric risks and danger signs. This knowledge, however, was not associated with preparation for birth or with women's use of institutional delivery.

These findings raise questions about the utility of focusing on danger signs and risk awareness in community-level interventions to promote women's use of professional maternity care. Instead, such interventions might be better focused on promoting household preparations for accessing skilled maternity care during all births and on catalyzing changes in social and gender norms related to birth preparedness, including increasing the involvement of male partners, which other research has highlighted as important (Iliyasu, Abubakar, Galadanci, & Aliyu, 2010; Kakaire et al., 2011; Mullany et al., 2007).

Similarly, based on the study findings, we suggest that ANC providers’ limited time for counseling might be best devoted to advising on place of delivery and specific preparations that should be made for any birth—normal or complicated. Almost two decades have passed since the antenatal risk screening approach has been discredited, and it is widely agreed that every birth should be attended by a skilled health professional (Campbell & Graham, 2006). Nevertheless, many women who attend antenatal care are not advised to deliver at a health facility. In our sample of women with recent births, fully 50% reported that they were not advised on any preparation for childbirth (i.e., setting aside money, arranging for transport, discussing and agreeing on plans with family members, etc.) during any of their ANC visits, and 44% reported that they were not advised to deliver in a health facility. The findings from our study suggest that focusing on risks and danger signs during ANC counseling may have little or no effect on routine care-seeking. The findings also raise questions about whether focusing on risks and danger signs may have the unintended effect of reinforcing outdated perceptions that home births are safe for “normal” pregnancies and deliveries.

Finally, we suggest that women's planning for and use of maternal health services may not be motivated by fear or concerns about risks—a finding that is consistent with conclusions from a meta-analysis of more than 350 HIV prevention interventions, which concluded that interventions designed to induce fear were not effective, compared with interventions that were designed in accordance with theories of reasoned action and planned behavior (Albarracín, Durantini, & Earl, 2006). Focusing on birth preparedness can offer women and their families specific, concrete actions they can take to ensure access to professional care during childbirth, whereas focusing on risks and danger signs does not appear to achieve the same results.

Consistent with these findings, it is worth noting that simply knowing pregnancy risks and danger signs will not be sufficient to overcome the formidable barriers posed by large geographic distances and limited means of transport—particularly in contexts where the limited availability and quality of basic obstetric care at primary health care facilities result in the practice of “bypassing” local health facilities because of the poor quality of care available (Kruk et al., 2009). Indeed, in such settings, advance decision-making, planning, and preparation for institutional delivery offer the only means of accessing such care when labor begins.

## ACKNOWLEDGMENTS

The authors thank Abigail Greenleaf for her review of the literature on birth preparedness, as well as Evelyn Landry, EngenderHealth, and Mary Ellen Stanton and Erin Mielke for reviewing and commenting on drafts of this article.

## FUNDING

This study was funded through the U.S. Agency for International Development (USAID), under associate cooperative agreement GHS-A-00-07-00021-00. Views expressed here do not necessarily reflect those of USAID.
